# A rare case of small intestinal metastasis from lung cancer

**DOI:** 10.1002/ccr3.6666

**Published:** 2022-11-27

**Authors:** Haitham Rejab, Sami Fendri, Ayman Trigui, Bassem Abid, Majdoub Youssef, Salma Ketata, Hazem Ben Ameur, Salah Boujelbene

**Affiliations:** ^1^ Department of general and digestive surgery, Department of anesthesia Habib Bourguiba Hospital Sfax Tunisia

**Keywords:** bowel obstruction, colic metastasis, lung cancer, surgery

## Abstract

We report the case of a 61‐year‐old male patient who was admitted with abdominal pain, vomiting and constipation. He had a past medical history of epidermoid lung cancer .computed tomography revealed distended stomach with mural bowel thickening. It was peroperatively two small‐bowel metastasis from lung cancer that we resect.

## Graphical abstract

Our case demonstrates that in case of acute bowel obstruction in a patient who had not undergone abdominal surgery previously, some diagnosis should be considered. In addition to colorectal tumor, bowel's tumor, and internal hernia, we should think of bowel metastasis that is a rare case, especially in a patient with lung cancer history.

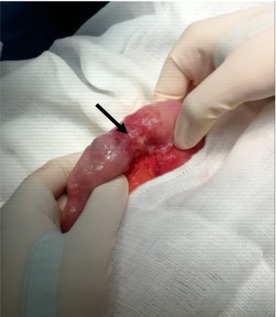

## 1 CASE PRESENTATION

**FIGURE 1:**
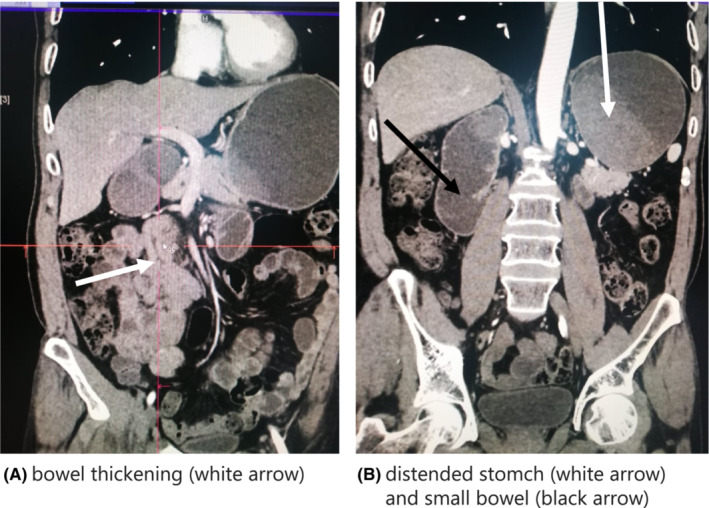
(A). Bowel thickening (white arrow). (B). Distended stomach (white arrow)

**FIGURE 2:**
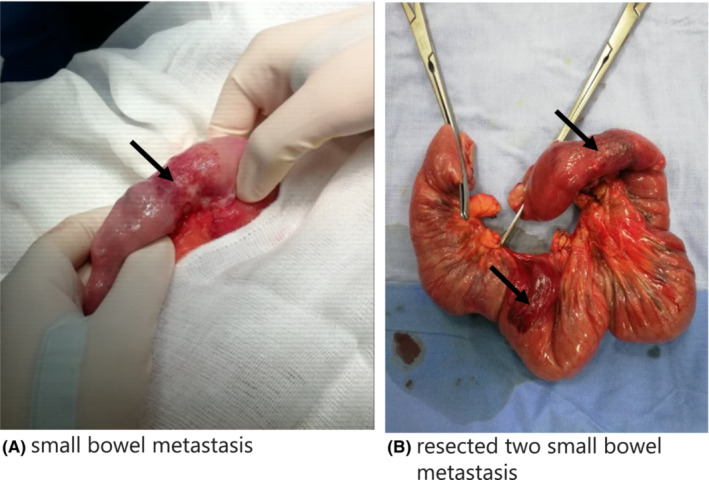
(A). Small bowel metastasis and small bowel (black arrow). (B). Resected two small bowel metastasis

A 61‐year‐old man presented to our emergency department with a complaint of a 3 days duration of lower abdominal pain, which had gradually migrated to epigastric area, vomiting, and constipation. The diagnosis of small bowel obstruction was suspected. Notably, he had a past medical history of epidermoid lung cancer, diagnosed 1 year prior. He was thought to be in remission following chemotherapy. Upon hospital admission, he underwent computed tomography (CT) of the abdomen and pelvis with contrast. Images revealed distended stomach with mural bowel thickening (Figure 1A,B). Midline incision laparotomy surgery with partial resection of the small intestine was performed. The tumor had invaded two parts of the small intestine (Figure 2A,B). There was no necrosis or ischemia of proximal bowel that was only distended. Pathology confirmed the diagnosis of small bowel metastasis from primary lung epidermoid carcinoma. The postoperative course was uneventful but the patient died 1 year later after lung cancer recurrence.^1^, ^2^

Even though rare, small bowel metastasis should be considered in the differential of patients with small bowel obstruction and history of lung cancer.

## AUTHOR CONTRIBUTIONS

Haitham Rejab operated the patient and wrote the manuscript. Sami Fendri operated the patient and searched bibliography. Ayman Trigui wrote the manuscript. Bassem Abid wrote the manuscript. Majdoub Youssef and Salma Ketata searched bibliography. Hazem Ben Ameur and Salah Boujelbene verified the manuscript before submission.

## CONFLICT OF INTEREST

None declared.

## ETHICAL APPROVAL

Personal data have been respected.

## CONSENT

Written informed consent was obtained from the patient to publish this report in accordance with the journal's patient consent policy.

## Data Availability

Personal data of the patient were respected. No data are available for this submission.
